# Hydraulic Optimization of Shaft Hydropower Plant Intake Configurations Using 3D-CFD: the At-Bashy Case, Kyrgyzstan

**DOI:** 10.1007/s00267-026-02535-9

**Published:** 2026-06-30

**Authors:** Bertalan Alapfy, Daniel S. Hayes, Moritz Roth, Nils Rüther

**Affiliations:** 1 Chair of Hydraulic Engineering, School of Engineering and Design, Technical University of Munich, Munich, Germany; 2 BOKU University, Department of Ecosystem Management, Climate and Biodiversity, Institute of Hydrobiology and Aquatic Ecosystem Management, Vienna, Austria

**Keywords:** Downstream fish guidance, Hydraulic-ecological optimization, Parametric hydraulic design, Sustainable hydropower, URANS free-surface modeling, Vertical intake hydraulics

## Abstract

The integration of shaft hydropower plant (SHPP) modules into existing weir structures offers a compact and potentially environmentally compatible solution for low-head hydropower generation. However, site-specific geometric constraints and operational variability can substantially affect intake hydraulics and, consequently, ecological performance. This study presents a structured three-dimensional CFD-based hydraulic optimization framework applied to the At-Bashy demonstration site (Kyrgyzstan), representing a SHPP configuration integrated into an existing gated weir. Nine intake configurations were systematically evaluated by varying trash rack length (6–8 m) and submergence above the rack (2.00–2.85 m) while maintaining constant turbine and bypass discharges. Fish-relevant hydraulic indicators were quantified within defined near-rack volumes of interest using time-averaged metrics. In addition to mean velocity components, tail-based indicators of extreme downward-directed velocities and statistical measures of free-surface variability were assessed. Results demonstrate that increasing submergence consistently reduces extreme downward velocities and homogenizes the near-rack flow field. Increasing rack length primarily lowers average downward deflection. Strong upstream flow asymmetry caused by geometric constraints induces localized flow separation and heterogeneous vertical flow velocity cores, highlighting the importance of site-specific hydraulic assessments. A rack length of 7 m combined with operational submergence levels between 2.35 m and 2.85 m provides a balanced compromise between ecological performance and structural-economic feasibility. The study confirms that SHPP intake optimization cannot rely on generalized empirical rules but requires site-adapted numerical evaluation. The presented CFD-based framework provides a transparent and transferable methodology for hydraulic optimization of SHPP intake configurations.

## Introduction

The rapid proliferation of small hydropower facilities worldwide has emerged as a promising avenue for renewable energy generation (Couto and Olden 2018; Jorde et al. 2022), yet it poses significant challenges to aquatic ecosystems, particularly by obstructing fish movements (Couto et al., 2021; He et al., 2024). As the number of these installations increases to meet growing energy demands, their cumulative impact on river connectivity and fish migration has raised urgent concerns about environmental sustainability (Lange et al., 2018; Baird et al., 2025). Bar racks with large clearances do not pose a behavioral barrier to fish, potentially leading to turbine entrainment of downstream-migrating individuals, which can cause injuries or even death (Pracheil et al., 2016; Radinger et al., 2022). To address these issues, there is a pressing need for hydropower solutions that prioritize fish protection while maintaining energy efficiency and economic feasibility.

The shaft hydropower plant (SHPP) concept was developed as an integrated low-head solution aimed at reconciling ecological compatibility with economic feasibility (Rutschmann et al., 2013). The complete electromechanical unit is installed within a vertical, rectangular shaft and operates fully submerged under upstream water-level conditions. Water approaches the intake horizontally at riverbed level and passes through horizontally arranged fine bar rack units with narrow bar clearance (technically feasible range between 10 mm and 20 mm), flush with the riverbed and covering the rectangular intake opening. The flow is directed vertically downward into the shaft, driving the submerged turbine-generator unit, and is discharged through a draft tube below tailwater level. This compact integration of intake, turbine, and draft tube reduces above-ground construction, improves flood resilience, and supports sediment continuity compared to conventional diversion schemes. The fine bar rack is intended to serve as a physical and behavioral barrier, preventing fish from being drawn into the turbine shaft (Sepp et al., 2016). Low normal velocities towards the trash rack further reduce the risk of fish entrainment. Downstream-migrating fish are instead guided toward site-specific bypass systems integrated into the vertical gate at the downstream end of the intake. These bypasses may be positioned near the water surface, at trash-rack level, or both, ensuring hydraulic accessibility and safe passage to the tailwater (Schäfer et al., 2016; Geiger et al., 2016). Proper geometric and hydraulic conditions support partial fish protection and downstream guidance, even for fish smaller than the bar clearance, where the screen functions not as a physical barrier but as a behavioral one. However, incorrect geometric and hydraulic arrangements of fine bar screens can cause significant harm to fish. The system’s effectiveness largely depends on behavior-based guiding effects, which require positive interaction between the screen, bypass setup, and flow conditions (Geiger et al., 2018).

Despite the conceptual ecological advantages of the SHPP, downstream fish passage remains a key concern. Initial experimental studies suggest that the combination of fine bar screens and vertical flow deflection could provide an effective barrier, guiding fish towards bypass routes (Geiger et al., 2016).

Still, field investigations have reported fish passage through the rack and turbine-related injuries (Knott et al., 2023a; Funk et al., 2024). However, the hydraulic boundary conditions during these monitoring campaigns were not fully quantified, given the lack of structured spatial flow measurements upstream of the rack. Also, site-specific shortcomings were identified, including significant damage to the trash rack during the experiments. Consequently, the hydraulic mechanisms leading to fish entrainment remain partly unresolved. These observations highlight the necessity for a hydraulically controlled, parameter-based assessment.

In its original prototype configuration, the SHPP intake was embedded within the river cross-section, allowing for a three-sided upstream approach flow that promoted relatively homogeneous velocity distributions across the rack surface. However, many potential sites for developing SHPPs do not permit such a configuration. When integrating the SHPP concept into existing weir structures, implementation is often constrained to a one-sided, channel-type approach. As demonstrated by Alapfy et al. (2024), under these conditions, intake hydraulics differ fundamentally from the three-sided prototype layout and are characterized by increased flow asymmetry, localized acceleration zones, and enhanced vertical deflection components. This situation requires dedicated optimization of rack geometry, intake area, and submergence.

At multi-purpose sites, additional complexity can arise from operational variability. Changes in upstream water level, for example, due to irrigation diversion, can directly alter the submergence above the trash rack, which in turn affects approach velocities, vertical deflection intensity, and surface flow uniformity. These hydraulic characteristics are particularly relevant for fish-protection performance, as strong downward-directed velocities may increase the probability of rack contact or entrainment. Pronounced surface non-uniformity can indicate heterogeneous approach flow and localized recirculation structures. Accordingly, the present study systematically investigates two primary design parameters under one-sided approach conditions: (i) trash rack length, which controls the effective intake area and is expected to influence the magnitude of vertical deflection, and (ii) submergence above the rack, which reflects operational headwater variability and directly affects the flow patterns at the intake. Building on a validated three-dimensional computational fluid dynamics (CFD) framework (Alapfy et al., 2024), the present study applies this model setup as a design tool to evaluate and optimize intake hydraulics for an SHPP case, where construction will be based on the simulation outcomes.

Here, we study a representative one-sided SHPP configuration requiring site-specific hydraulic optimization. The present study evaluates the combined influence of effective intake area and intake submergence on near-rack velocity characteristics and surface flow uniformity under operationally relevant boundary conditions with respect to both fish ecological and hydraulic performance within site-specific technical and economic limits. It is hypothesized that enlarging the effective intake area and intake submergence will reduce downward-directed velocities and homogenize the spatial distribution of the approach flow, thereby improving hydraulic guidance conditions and enhancing overall hydraulic performance. To test this hypothesis, a structured three-dimensional numerical investigation is conducted.

The specific objectives of this study are: (i) to evaluate how trash rack length (6–8 m) affects near-rack velocity indicators under one-sided approach flow conditions; (ii) to evaluate how operational submergence depth (2.00–2.85 m) affects those same indicators; and (iii) to identify an intake configuration that balances hydraulic-ecological performance with structural and economic constraints at the At-Bashy demonstration site.

## Materials and Methods

### Case Study Site and Planned Hydropower Layout

**Fig. 1:**
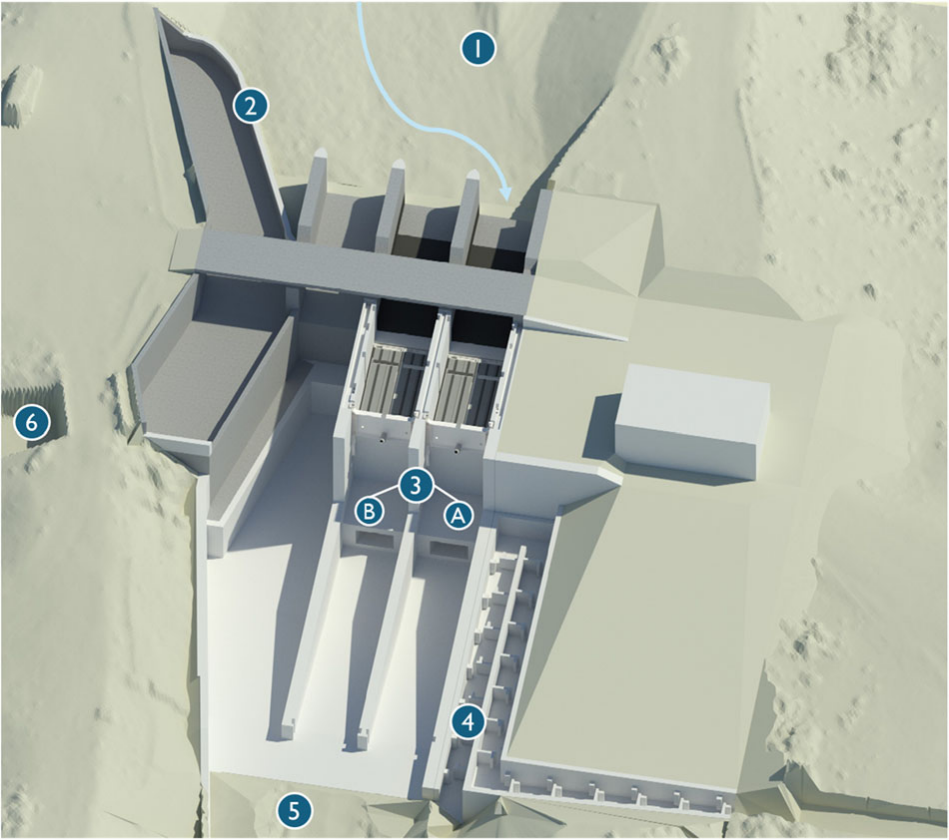
3D-rendering of the final project layout, with existing structures in dark grey and planned structures in light grey. (1) upstream riverbed, (2) irrigation diversion channel, (3) shaft hydropower plant (SHPP) with (A) left module and (B) right module, (4) vertical slot fish pass, (5) downstream riverbed, (6) irrigation diversion channel. The arrow indicates the flow direction

**Fig. 2:**
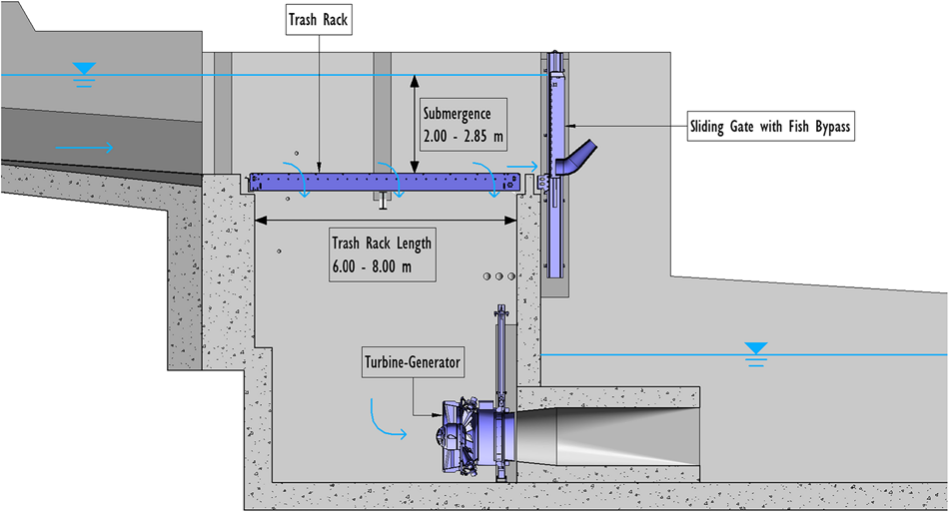
Schematic longitudinal section through the analyzed orographic left SHPP module

The study site is a planned hydropower plant located at the At-Bashy River, a tributary to the Naryn River, 8 km west of Ak Muz village in the At-Bashy district of the Kyrgyz Republic (41° 13′ 48.9″ N, 76° 13′ 50.9″ E). The hydropower project concept includes the refurbishment of an aged, gated weir structure built in the 1970s for irrigation water diversion. The weir is equipped with three pressure segments and a fixed-crest side-overflow wall on the orographic right side. With the SHPP concept, the existing weir structure’s gross head of almost 8 m can be utilized without requiring a diversion channel. Therefore, the SHPP modules are integrated directly downstream of the weir gates (Figs. 1, 2). The operational water level is then regulated primarily by the automatically adjustable turbine discharge and secondarily by the vertical slide gates, which can be raised for trash-rack flushing and lowered for flood discharge (Alapfy et al., 2025).

In the presented case study, two identical SHPP modules are planned to be integrated into the existing weir structure (Fig. 1), each equipped with a turbine-generator unit with 1,250 mm runner diameter and a design flow of Q_T_ = 8.5 m^3^/s. The rectangular intake openings of the SHPP modules are each covered by two identical trash-rack-units with a bar clearance of 15 mm. A rectangular fish bypass opening (0.5 × 0.5 m) is integrated into the bottom of the vertical slide gates; from the inlet (upstream) side, it smoothly transforms into a cylindrical riser pipe with an opening diameter of 0.3 m on the outlet (downstream) side, with a permanent flow release of approximately 0.25 m^3^/s. The objective of this design is to lead fish horizontally across the rack, above the SHPP intake opening, towards the bypass opening, where the streamwise flow velocity is gradually increased, leading fish through the riser pipe and plunging into the tailrace channel downstream of the SHPP draft tubes, where the water depth is at least 2.5 m or more. The detailed hydraulic analysis of this unconventional fish-bypass design is not part of the presented study due to scaling reasons. In turn, this system will be analyzed, and, if needed, optimized separately as part of the Hydro4U project’s monitoring program.

Given the objective to integrate the SHPP modules into the existing weir structure, the following site-specific limitations must be considered for the dimensioning of the SHPP intakes, especially regarding the two hydraulically most relevant variables: (i) the intake area and (ii) the submergence of the trash rack. First, the clear width of the weir fields is 4.8 m, which automatically sets the width of the SHPP intakes. Thus, the intake area can only be varied by adjusting the length of the intake opening and thus the length of the trash rack modules. Given further economic limitations (i.e., equipment costs, particularly the costs for trash-rack modules), the maximum cost-viable length of the intake opening is set at 8.0 m. Secondly, the facility’s main purpose will remain to be the diversion of irrigation water. From May to mid-September, up to 15 m^3^/s will be diverted for irrigation, necessitating a higher headwater level for water to flow over the side-overfall crest. From mid-September to March, the entire inflow can be used for hydropower generation, so that the headwater level will be reduced to the top level of the side-overfall crest to lead all of the available flow towards the SHPP intakes. While the At-Bashy SHPP aims to increase the total head for maximizing energy production, the discharge capacity of the upstream side-overfall wall, over which the irrigation water is diverted, needs to be considered together with the height of the existing upstream embankment dams. Accounting for flood safety and continued irrigation water diversion, the maximum possible increase of the side overfall wall is 0.35 m. Given the site-specific constraints, the following submergence values above the SHPP intake are relevant for the case study: 2.00 m reflects the current condition without modifications to the upstream irrigation diversion; 2.35 m corresponds to an increased top level of the irrigation diversion wall during winter (headwater level equal to the wall’s top, no overflow); and 2.85 m applies to the irrigation season with a modified wall (headwater approximately 0.5 m above the wall).

### Fish Ecological Assessment

A semi-quantitative electrofishing survey was conducted in October 2022 to characterize the fish community in the At-Bashy River in the vicinity of the proposed SHPP. Sampling was performed using backpack electrofishing equipment (Honda FEG 1500, 1.5 kW) in river sections both upstream and downstream of the existing weir. In each section, all major habitat types were targeted proportionally, including riffles, runs, pools, bays, and shoreline areas, to ensure representative coverage of available mesohabitats (Alapfy et al., 2023).

The survey confirmed the presence of three native fish taxa in the At-Bashy River (Alapfy et al., 2023): loach (*Triplophysa* sp.), osman (*Diptychus* sp.), and snow trout (*Schizothorax* sp.). These taxa are typical of high-altitude, fast-flowing Central Asian mountain rivers (Kustareva and Naseka 2015; Karimov et al., 2025). Mean total lengths (±standard deviation) were highest for snow trout (104.9 ± 35.5 mm), followed by osman (97.1 ± 25.3 mm) and loach (79.5 ± 19.1 mm), reflecting differences in growth patterns and life-history strategies among the fish taxa.

All three taxa occurred both upstream and downstream of the weir. Overall, the fish community exhibited evidence of natural reproduction across the sampled reaches. The consistent presence of young-of-the-year individuals in all sections indicated successful local spawning and recruitment for each species. However, length-frequency distributions across the sampled reaches suggested potential impacts of the weir on population structure. Notably, adult fish densities were lower upstream of the barrier compared to downstream sections. The highest overall fish densities of subadult and adult fish were recorded at the most downstream sampling sites, which may be used as winter habitat (Alapfy et al. 2023). These observations highlight the significance of longitudinal river connectivity for migratory movements in this system. The weir appears to partially impede upstream spawning migrations of adult fish, especially snow trout, which exhibit potamodromous behavior in mountain rivers (Omonov et al., in review), thereby limiting stock replenishment and reducing population densities upstream. Such fragmentation effects underscore the need for effective fish passage solutions in the optimized intake configurations evaluated in this study.

### 3D Model Construction

No as-built drawings of the existing hydraulic structures were available. Therefore, a detailed 3D-as-built model of the structure and the relevant upstream and downstream river stretches was created using Structure-from-Motion (SfM) photogrammetry (Westoby et al. 2012). Data acquisition was conducted under dry channel conditions, enabling complete surface capture. Prior to flight operations, the survey boundary (1.25 km²) was delineated in QGIS and imported into DJI Pilot 2 for automated mission planning and optimized flight path generation. A total of 3,398 images were acquired during a flight time of approximately 1 h 33 min. Image acquisition was performed using a DJI Matrice 300 RTK equipped with a DJI Zenmuse P1 (35 mm fixed focal length) at an altitude of 120 m above ground level. To ensure radiometric consistency and geometric stability, aperture and shutter speed were maintained at constant values throughout the mission. ISO sensitivity was set to automatic mode to compensate for variable illumination conditions while preserving consistent exposure parameters. For georeferencing, 32 ground control points (GCPs) were evenly distributed across the study area. Their coordinates were measured using a real-time kinematic Global Navigation Satellite System (RTK-GNSS) base–rover configuration based on the Emlid Reach RS system. The base station provided real-time correction data to the rover, enabling centimeter-level positioning accuracy. Photogrammetric processing was carried out using Agisoft Metashape Professional Edition (Version 1.5.2). All images were imported, and low-quality images were excluded prior to processing. The workflow comprised image alignment, sparse and dense point cloud generation, and derivation of digital elevation models (DEMs) and textured 3D meshes. The dataset was referenced to the WGS84/UTM Zone 43 N coordinate system. GCP coordinates were imported and manually assigned to the corresponding image observations to optimize spatial accuracy. The final model achieved residual errors in Easting, Northing, and elevation of less than 3 cm relative to the reference coordinate system.

The final outcomes of this workflow included a georeferenced orthophoto as well as a digital elevation model (DEM) of the site, as well as a point cloud of the existing structures, which was integrated into a building information model that contains the geometry of the existing structure’s components as well as the existing terrain contours. This information formed the basis for the subsequent engineering design of the refurbishment works and the integration of the SHPP modules into the weir structure in combination with a fish migration facility (Fig. 1).

The present study focuses on the hydraulic-numerical analysis of the orographic left SHPP module (Fig. 1, component 5 A), as that is the one to be built and commissioned first within the framework of the Hydro4U project.

### Hydraulic-Numerical Methods

#### Model Setup

The numerical model represents the approach channel upstream of the gated weir and the intake reach above the shaft power plant module. The intake is covered by two identical, rectangular horizontal trash rack modules, each having a width of 2.4 m. For the numerical model setup, their main components need to be differentiated: the non-permeable steel frame and the permeable fine grid. The fish downstream bypass is located at the downstream end of the trash rack system, with a bottom opening (see “Case Study Site and Planned Hydropower Layout”).

For the 3D-CFD simulations, the commercial software FLOW-3D^®^ HYDRO (Version 2024R2; Flow Science, Inc., Santa Fe, NM, USA) was used. The flow was solved using the transient Reynolds-averaged Navier–Stokes equations with the RNG k–epsilon turbulence closure (Yakhot and Orszag, 1986) and a volume-of-fluid (VOF) free-surface formulation (Hirt and Nichols, 1981; Kahraman et al., 2020). The applied solver formulation corresponds to an unsteady Reynolds-averaged Navier–Stokes (URANS) approach, resolving transient large-scale flow dynamics while modeling turbulence statistically. Simulations were run in the following manner: For each case, an initialization simulation with a coarse mesh resolution, applying 0.12 m nominal cell size instead of 0.06 m in the area of interest, was run for 240 s. Using the software’s restart-simulation function, this preliminary result served as the initial condition for the subsequent production simulation, which was run for 90 seconds. Using this approach reduces total computation time, as the production simulation reaches a statistically steady mean state faster, since the initial condition already approximates the final state. Residual fluctuations persist due to turbulence modeling, free surface oscillations, and recirculation variability.

Given computational limitations with regard to the scale of the present study, it is not feasible to resolve the individual bar geometry of the trash rack modules. Thus, the permeable grid of the trash rack modules was modeled as a porous medium, applying the Forchheimer saturated drag model with constant drag coefficients A = 28125 1/m^2^ and B = 0.95 1/m for all simulations. The porosity, defined as the open volume divided by the total volume in each direction as well as the specific surface area, was determined based on the actual geometry of the trash rack (i.e., number of bars, their length, depth, thickness, and spacing). For the representation of the turbine runner in motion, the fan model properties were set in accordance with the planned configuration of the actual turbine for this project as follows: spin rate, 50.27 rad/s (480 rpm); thickness, 0.5 m; blade tip thickness, 0.01 m; and number of blades, 3. The numerical model setup applied here — including the URANS-VOF formulation, RNG k–ε turbulence closure, and porous media representation of the trash rack using the Forchheimer saturated drag model — has been validated against physical model measurements for a hydraulically comparable shaft hydropower plant intake in Alapfy et al. (2024), demonstrating satisfactory agreement in head loss and near-rack velocity distribution. That validation study established the introduced drag coefficients used here without modification.

Nine intake configurations were evaluated by varying two geometric-hydraulic parameters – the trash rack length and submergence above the trash rack (Fig. 2) – while keeping the intake width constant. The intake width is fixed by the existing weir field geometry (4.8 m clear width) and is covered by two identical rack modules. Each module has a structural width of 2.4 m and an effective net rack width of 2.0 m, resulting in a total effective intake width of 4.0 m for all configurations. Regarding the trash rack length (TR), we tested lengths of 6 m, 7 m, and 8 m. Submergence above the trash rack (h) was modeled for 2.00 m, 2.35 m, and 2.85 m. These values correspond to operationally relevant headwater conditions at the study site (see “Case Study Site and Planned Hydropower Layout”).

A Cartesian coordinate system aligned with the FLOW-3D grid is used throughout. The x-direction is the main horizontal approach-flow direction, the y-direction is lateral, and the z-direction is aligned with gravity (positive upward). Accordingly, vertical velocity towards the trash rack is negative z. Velocity components are reported as *u* (x), *v* (y), and *w* (z). The lateral velocity component (*v*) was inspected during post-processing. Within the defined volumes of interest above the trash rack (see below), *v* was consistently small compared to *u* and *w* and did not exhibit systematic trends across the tested configurations. Because the primary research questions concern downward-directed velocities toward the rack and streamwise transport toward the bypass, the analysis focuses on *u* and *w*.

**Fig. 3:**
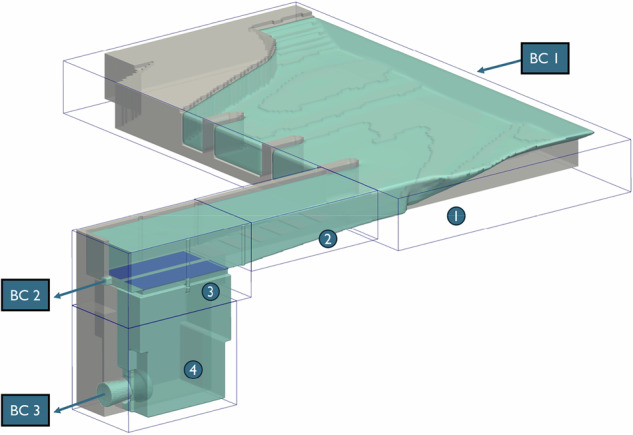
CFD Model setup showing model structure, mesh blocks (numbered from upstream to downstream, 1–4) and boundary conditions (BC). BC 1: Inlet with constant water level (2.00 m/2.35 m/2.85 m), BC 2: Outlet with constant flow (0.25 m^3^/s), BC 3: Outlet with constant flow (8.5 m^3^/s). The solid model domain is shown in grey, the water volume is shown in opaque turquoise, the fine grid of the trash rack modules, modeled as porous media, in blue. The solid volume is clipped along the longitudinal section of the SHPP module for improved visibility. See Table 1 for further details

A structured Cartesian mesh with local refinement was applied. The resolution of the mesh blocks (Fig. 3) is refined stepwise from the upstream area within block 1 (nominal cubic cell size of 0.24 m) over the approach channel within block 2 (nominal cubic cell size of 0.12 m) to the intake region of interest within blocks 3 and 4 (nominal cubic cell size of 0.06 m), selected based on the grid sensitivity analysis. Because FLOW-3D’s automatic vertical cell distribution may lead to a small deviation from the nominal cell height, post-processing regions were defined by geometric elevations relative to the trash rack plane (z = 0), rather than assuming an integer number of grid layers. The structured mesh is resolved in the highest resolution of 0.06 m in the mesh blocks above and below the trash rack, while the mesh resolution gradually decreases towards the upstream direction by a factor of two, resulting in a cell size of 0.12 m in the approach channel, and 0.24 m in the riverbed upstream of the existing weir. The boundary conditions are as follows:

- Constant water level at the upstream boundary, corresponding to the targeted operational water level above the intake (2.00 m, 2.35 m, 2.85 m).
- Constant discharge through the fish-bypass (0.25 m^3^/s) in the positive x-direction.
- Constant discharge through the draft tube cone (8.5 m^3^/s) in the positive x-direction.
- Irrigation diversion was not modeled.

The mesh structure and the model boundary conditions of the production simulations are summarized in Table 1.

**Table 1:** Summary of computational mesh configuration and boundary conditions for production simulations

Mesh block	Region / Purpose	Cell size (m)	Dimensions L × W × H (m)	Boundary condition
MB 1	Upstream river reach; captures approach flow and weir inlet pier	~0.24	18.84 × 35.0 × 3.1	BC 1 (x-min): Constant fluid elevation (2.00, 2.35, or 2.85 m above rack)
MB 2	Horizontal approach channel through weir field; grid planes aligned with MB 1	~0.12	10.16 × 5.76 × 3.1	—
MB 3	Upper HPP intake; trash rack modules (porous media) and fish bypass opening; grid planes aligned with MB 2	~0.06	9/10/11 × 5.76 × 5.0	BC 2 (x-max): Constant flow rate 0.25 m³/s (fish bypass, fluid fraction = 1)
MB 4	Lower HPP intake; turbine-generator unit and draft-tube cone; grid planes aligned with MB 3	~0.06	9/10/11 × 5.76 × 7.0	BC 3 (x-max): Constant flow rate 8.5 m³/s (draft tube, fluid fraction = 1)

MB 3 and MB 4 lengths increase by 1 m per additional meter of trash rack length

#### Post-Processing and Evaluation Metrics

**Fig. 4:**
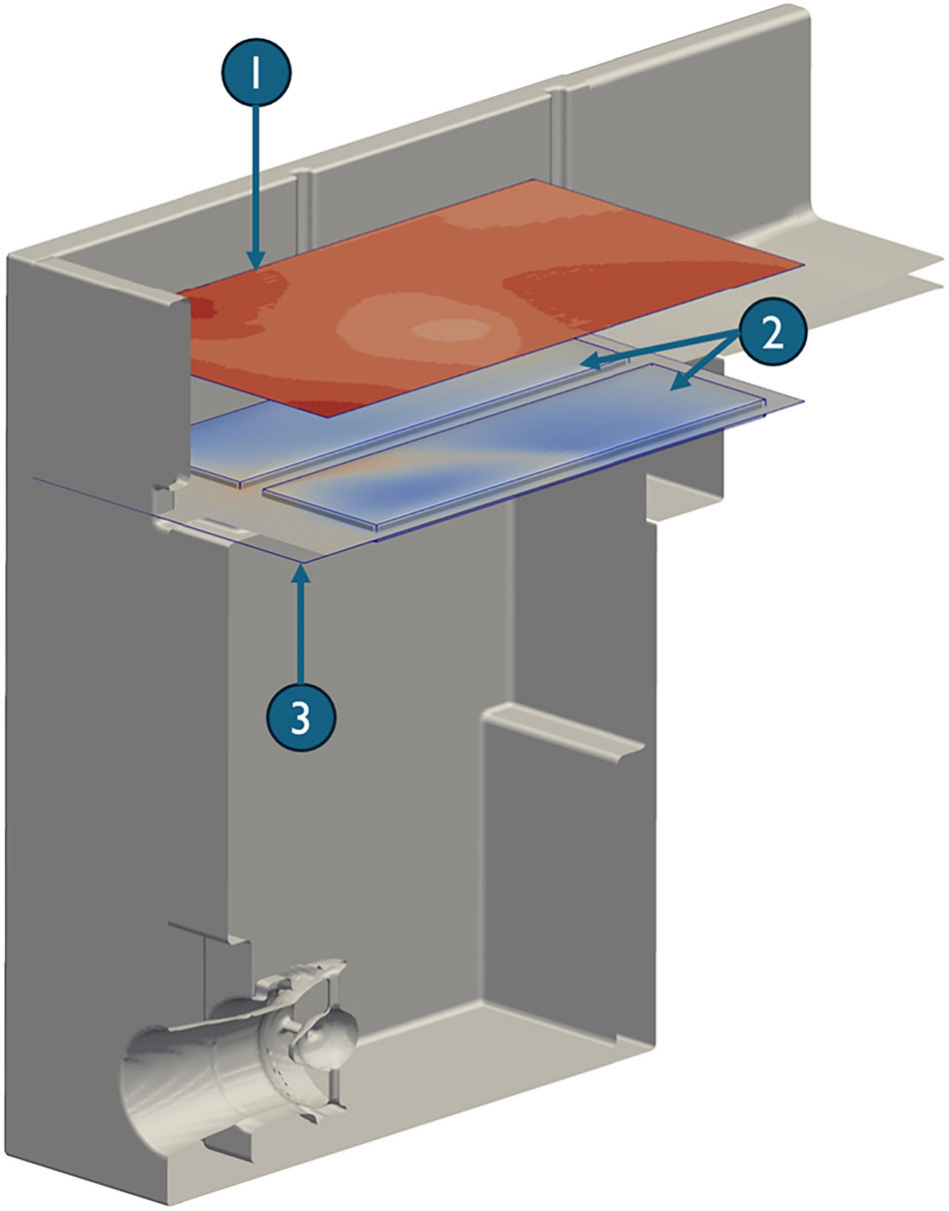
Evaluation objects within the model domain: (1) near-surface z-slice for visualizing free-surface elevation (FSE) variability, (2) left- and right-side volume-of-interest (VOI) boxes above the permeable areas of the trash-rack modules, (3) near bottom z-slice above the trash-racks. The solid part of the model domain is clipped for improved visibility of the evaluation objects

The primary evaluation focus is the flow field above the trash rack, because this region governs exposure of fish to downward velocities towards the rack and the capacity to guide fish downstream towards the bypass. Two volume-of-interest (VOI) boxes were defined above the permeable areas of the left and right trash rack modules (Fig. 4). Metrics are reported for the combined evaluation, where samples from both VOIs are pooled. Within each VOI, *u* (streamwise velocity) and *w* (vertical velocity) values were exported for all sampled cells. The following indicators were computed:

- Mean *u*: mean streamwise velocity in the VOI.
- Mean *w*: mean vertical velocity in the VOI (negative values indicate flow towards the trash rack).
- P5 *w*: 5th percentile of *w* (represents stronger downward velocities because w is negative towards the rack). The 5th percentile was selected to capture hydraulically relevant extreme downward velocities while remaining robust against single-cell outliers; lower thresholds would overemphasize numerical noise, whereas higher thresholds would increasingly reflect bulk behavior rather than localized exposure zones.
- TailMean5 *w*: mean of the lowest 5% of *w*-values (tail-average indicator).
- Ratio = abs(Mean *u*)/abs(Mean *w*) as a compact measure of streamwise transport versus downward deflection.

The combined use of mean-based and tail-based indicators allows differentiation between overall loading and localized extreme exposure zones, which may be particularly relevant for fish interaction with the rack. For visualization of the simulation results, a slice normal to the z-axis was used at a height of 0.125 m above the trash rack, corresponding to the middle of the height of the VOI-boxes.

Secondly, to characterize surface non-uniformity above the intake, free-surface elevation (FSE) was evaluated over the intake area using a slice slightly below the mean free surface (Fig. 4). Free-surface variability was quantified using:

- FSE_P95_minus_P5: interquantile range (P95 minus P5) of FSE.
- FSE_std: spatial standard deviation of FSE.
- Qualitative free-surface visualizations use a representative late-time snapshot, and the reported FSE statistics are computed from the same snapshot for consistent cross-case comparison.

As the transient formulation of the numerical solver and free-surface dynamics still produce residual temporal variability in the resolved fields (free-surface oscillations, recirculation variability, numerical noise), the quantitative comparison of both mean-based and tail-based indicators was conducted based on time-averaged VOI statistics over the final 60 s of each simulation, corresponding to 13 exported time steps at 5 s intervals. Qualitative flow visualizations use representative snapshots of the last timestep of each production simulation.

#### Sensitivity Analyses and Domain Extent Study

Prior to evaluating the nine production configurations, three verification steps were conducted. Firstly, a VOI-height sensitivity assessment to determine a robust VOI height; secondly, a domain-extent study to confirm the upstream boundary distance does not unduly influence near-rack indicators; thirdly, a grid-sensitivity analysis to assess indicator sensitivity to intake-region cell size.

The hydraulic evaluation of fish-relevant flow conditions was based on a VOI defined directly above the trash rack. Downstream migrating, bottom-oriented fish taxa of the At-Bashy River (see “Fish Ecological Assessment”) are expected to primarily interact with the flow in the near-rack region (Svendsen et al. 2007; Ebel 2024; Funk et al. 2024); therefore, the vertical extent of this VOI represents an important modeling choice. To assess the sensitivity of the results to the selected VOI height, three VOI heights were evaluated: 0.20 m, 0.25 m, and 0.30 m above the trash rack. The analysis was performed for the configuration with a trash rack length of 6 m and a submergence of 2.0 m, which exhibits pronounced near-rack flow deflection. For each VOI height, time-averaged vertical velocity metrics were extracted over the final 60 s of simulation time.

A domain-extent study was conducted to assess the influence of upstream domain length on flow conditions approaching the intake. The objective of this analysis was to ensure that the imposed upstream boundary does not affect the approach flow field and, consequently, the near-rack flow metrics used for the comparative evaluation of intake configurations. The upstream domain length was systematically varied, while all other geometric, hydraulic, and numerical parameters were kept constant, using the intake configuration with 6.0 m trash-rack length and 2.85 m submergence. The evaluation focused on the same near-rack VOI metrics later used in the main analysis, including time-averaged vertical velocity indicators extracted over the final 60 seconds of simulation time.

Lastly, a grid sensitivity analysis was performed to evaluate the influence of spatial resolution on the near-rack flow metrics. Three structured grid resolutions were tested, with cubic cell edge lengths of 72 mm, 60 mm, and 50 mm in the region of interest, respectively. The analysis was conducted using the same intake configuration and VOI definition as for the domain extent study.

## Results

### Model Sensitivity Results

The sensitivity analysis for the three different VOI heights shows a slight reduction in downward velocity magnitudes with increasing VOI height, affecting both mean values and tail-based indicators (Table 2). This behavior is physically expected, as increasing the VOI height progressively includes regions farther away from the trash rack where downward flow components are weaker. Importantly, the relative trends and qualitative behavior of the evaluated metrics remain unchanged across the tested VOI heights. Thus, the VOI height above the trash rack was fixed to 0.25 m for all subsequent analyses and production results. This height represents a compromise between capturing near-rack flow effects while avoiding excessive sensitivity to very local rack-proximal features. In addition, it corresponds to the lower half of the fish bypass opening height and is, therefore, considered physically relevant for fish guidance assessment.

**Table 2:** Volume-of-interest (VOI) height sensitivity results (combined VOI; 13 time steps, totaling 60 s)

VOI height (m)	Mean *u* (m/s)	Mean *w* (m/s)	P5 *w* (m/s)	TailMean5 *w* (m/s)
0.20	0.70	−0.33	−0.73	−0.83
0.25	0.68	−0.33	−0.72	−0.82
0.30	0.70	−0.32	−0.72	−0.82

The domain-extent study assessed whether the upstream boundary distance affects the near-rack indicators in the region of interest. The shortest domain length variant (+0.0 m) reaches only until the upstream end of the SHPP intake channel, without capturing the pier of the existing weir field. As expected, the largest change occurs when extending the domain extent from +0.0 m to +10.0 m (Table 3), indicating that the asymmetric flow field caused by flow separation at the orographic right inlet pier significantly influences the near-rack flow. Extensions beyond +10.0 m produce comparatively smaller changes in the combined VOI indicators (Table 3). Considering the objective of this study as a relative comparison of intake configurations, and the fact that all configurations were simulated using the same upstream domain extent, an upstream domain length of +20.0 m was adopted for all production simulations.

**Table 3:** Domain-extent study results (combined volume-of-interest; 13 time steps, totaling 60 s)

Domain extent (m)	Mean *u* (m/s)	Mean *w* (m/s)	P5 *w* (m/s)	TailMean5 *w* (m/s)
+0.00	0.37	−0.30	−0.47	−0.50
+10.00	0.51	−0.31	−0.50	−0.54
+20.00	0.49	−0.31	−0.49	−0.54
+30.00	0.47	−0.31	−0.48	−0.51

The grid-sensitivity analysis compared three intake-region mesh resolutions (Table 4). Mean *w* is effectively unchanged across the tested meshes (staying constant at −0.33 m/s). Tail-based metrics associated with extreme downward velocities display a more pronounced sensitivity to grid refinement, particularly when transitioning from the coarsest grid (72 mm) to finer resolutions (60 mm, 50 mm). However, differences between the 60 mm and 50 mm grids are small and decrease monotonically, indicating convergence. To quantify this: TailMean5 *w* changes from −0.62 m/s (60 mm) to −0.65 m/s (50 mm), a difference of approximately 5%, indicating that the solution is approaching convergence. Given the substantially increased computational cost associated with further grid refinement and the negligible improvement in the evaluated metrics, a grid resolution of 60 mm was selected for all production simulations. Since all intake configurations were evaluated using the same grid resolution, remaining grid-related uncertainties affect all cases consistently and do not compromise the comparative assessment.

**Table 4:** Grid-sensitivity analysis results (combined volume-of-interest; 13 time steps, totaling 60 s)

Cell size (mm)	Mean *u* (m/s)	Mean *w* (m/s)	P5 *w* (m/s)	TailMean5 *w* (m/s)
72	0.39	−0.33	−0.54	−0.60
60	0.41	−0.33	−0.56	−0.62
50	0.46	−0.33	−0.58	−0.65

### Comparative Results for Nine Intake Configurations

**Fig. 5:**
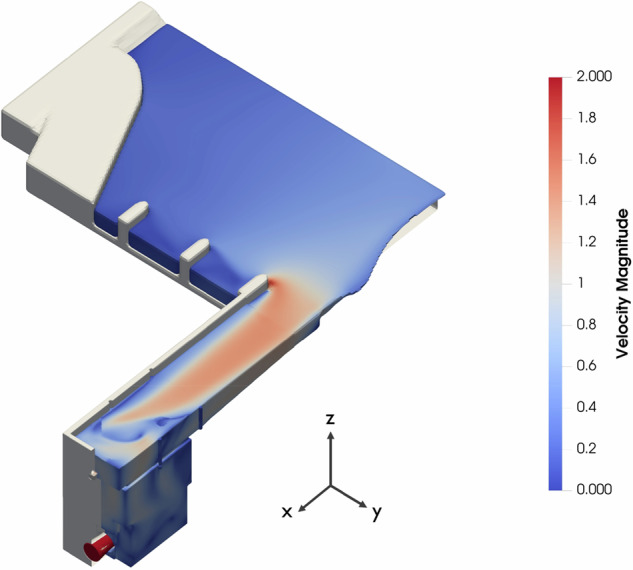
Exemplary 3D-snapshot of a production simulation (case 7.00 m long trash rack, 2.35 m submergence). Rendered for total velocity magnitude, showing heterogeneous velocity distribution at the fluid surface. Asymmetric approach flow and indications of flow separation are visible at the orographic right pier of the left weir field

**Fig. 6:**
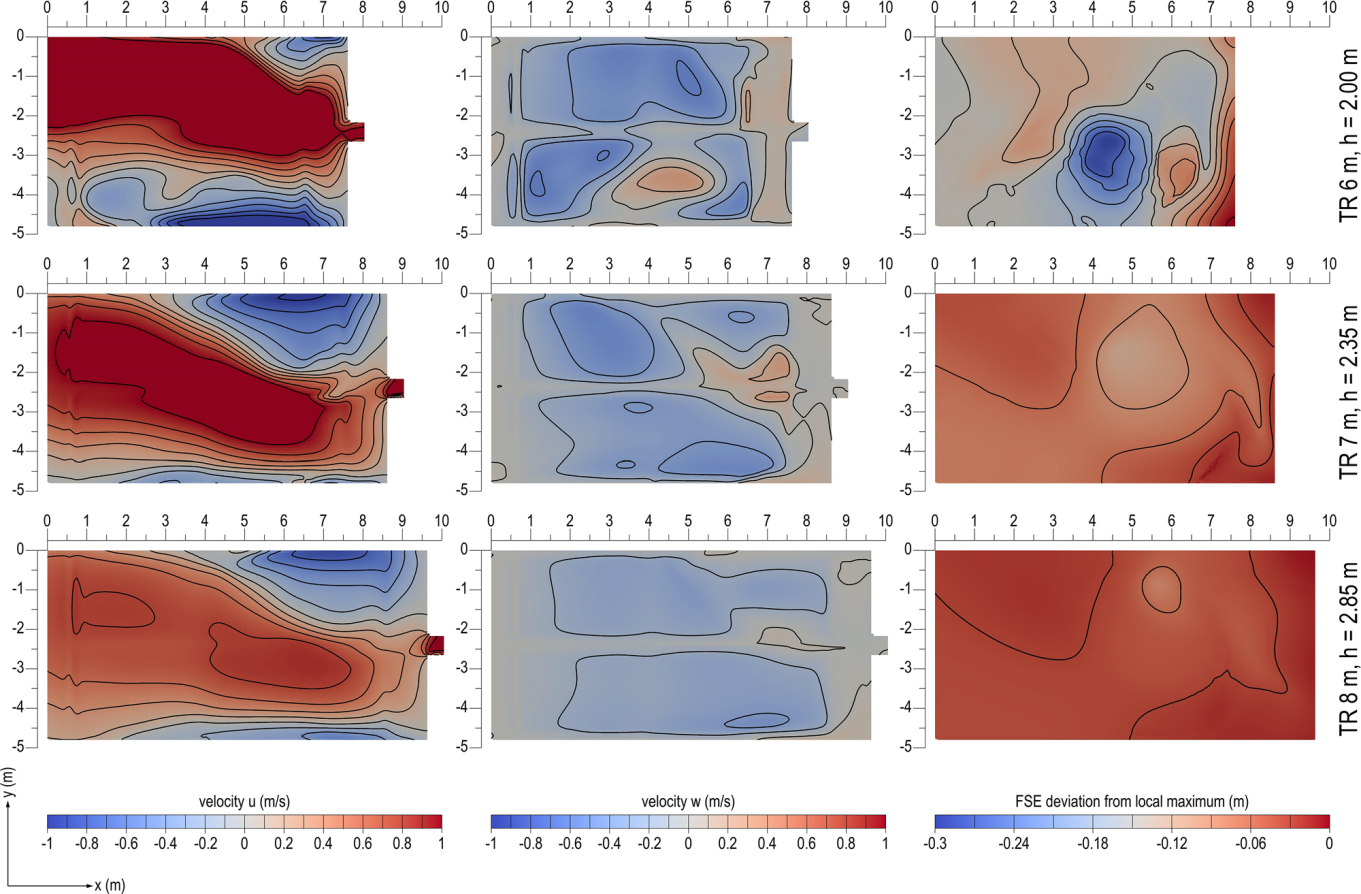
Snapshots at *t* = 90 s of streamwise velocity (*u*) (left column), vertical velocity (*w*) (middle column), and normalized free-surface elevation (FSE) deviation (right column) for three selected intake configurations (TR=trash rack length (m), h = submerged depth (m)). Flow velocity patterns show the z-slice at 0.125 m above the trash rack modules

Production simulations were performed for nine configurations combining three trash rack lengths and three submergence levels. At the At-Bashy weir, the approach flow upstream of the intake exhibits a distinct lateral asymmetry (Fig. 5). Under the simulated discharge conditions, the entire river flow is directed into the orographic left weir field, requiring a double deflection: first laterally toward the left weir field, then back toward the right upon passing the orographic right pier of that field. This double deflection induces a flow separation zone on the orographic right side of the approach channel, which persists downstream and is carried through to the SHPP intake face, directly affecting the spatial distribution of near-rack velocities. This asymmetry is reflected in the spatial distribution of velocity cores above the rack (Fig. 6).

For a specific submergence level, mean *u* remains relatively stable. At a submergence of 2.00 m, it ranges from 0.70 m/s at a trash rack length of 6.00 m to 0.75 m/s at a trash rack length of 8.00 m. When considering a fixed trash rack length, mean *u* decreases consistently as submergence increases. For instance, at a trash rack length of 6.00 m, mean *u* decreases from 0.70 m/s at a submergence of 2.00 m to 0.41 m/s at 2.85 m depth (Table 5).

**Table 5:** Production simulation results of the nine tested combinations of trash rack length and submergence depth (combined volume-of-interest (0.25 m), upstream domain extent +20 m, 60 mm intake-region mesh; results show time-averaged last 13 time steps, totaling 60 s)

Intake configurations									
ID	Trash rack length (m)	×	Submergence depth *h* (m)	Mean *u* (m/s)	Mean *w* (m/s)	TailMean5 *w* (m/s)	*u*/*w* ratio	FSE P95-P5 interquartile range (m)	FSE_std (m)
1	6	×	2.00	0.70	−0.33	−0.83	2.10	0.15	0.04
2	6	×	2.35	0.51	−0.33	−0.74	1.53	0.08	0.03
3	6	×	2.85	0.41	−0.33	−0.62	1.23	0.03	0.01
4	7	×	2.00	0.70	−0.29	−0.78	2.39	0.11	0.03
5	7	×	2.35	0.45	−0.29	−0.63	1.55	0.07	0.02
6	7	×	2.85	0.41	−0.29	−0.53	1.42	0.02	0.01
7	8	×	2.00	0.75	−0.27	−0.70	2.81	0.08	0.02
8	8	×	2.35	0.48	−0.26	−0.63	1.82	0.07	0.02
9	8	×	2.85	0.45	−0.26	−0.54	1.70	0.03	0.01

*FSE* = free-surface elevation

For a given submergence, increasing rack length reduces the magnitude of downward flow velocities. At 2.00 m submergence, for example, mean *w* decreases by 18% from −0.33 m/s (6.00 m) to −0.27 m/s (8.00 m). TailMean5 *w* shows a similar reduction in magnitude of 16% (Table 5). While mean *w* values show moderate variation across configurations, the tail-based metrics and spatial visualizations reveal pronounced localized extremes for low-submergence cases. Regions of strongly negative *w* coexist with areas of weakly positive vertical velocity, resulting in heterogeneous near-rack flow patterns (Fig. 4).

**Fig. 7:**
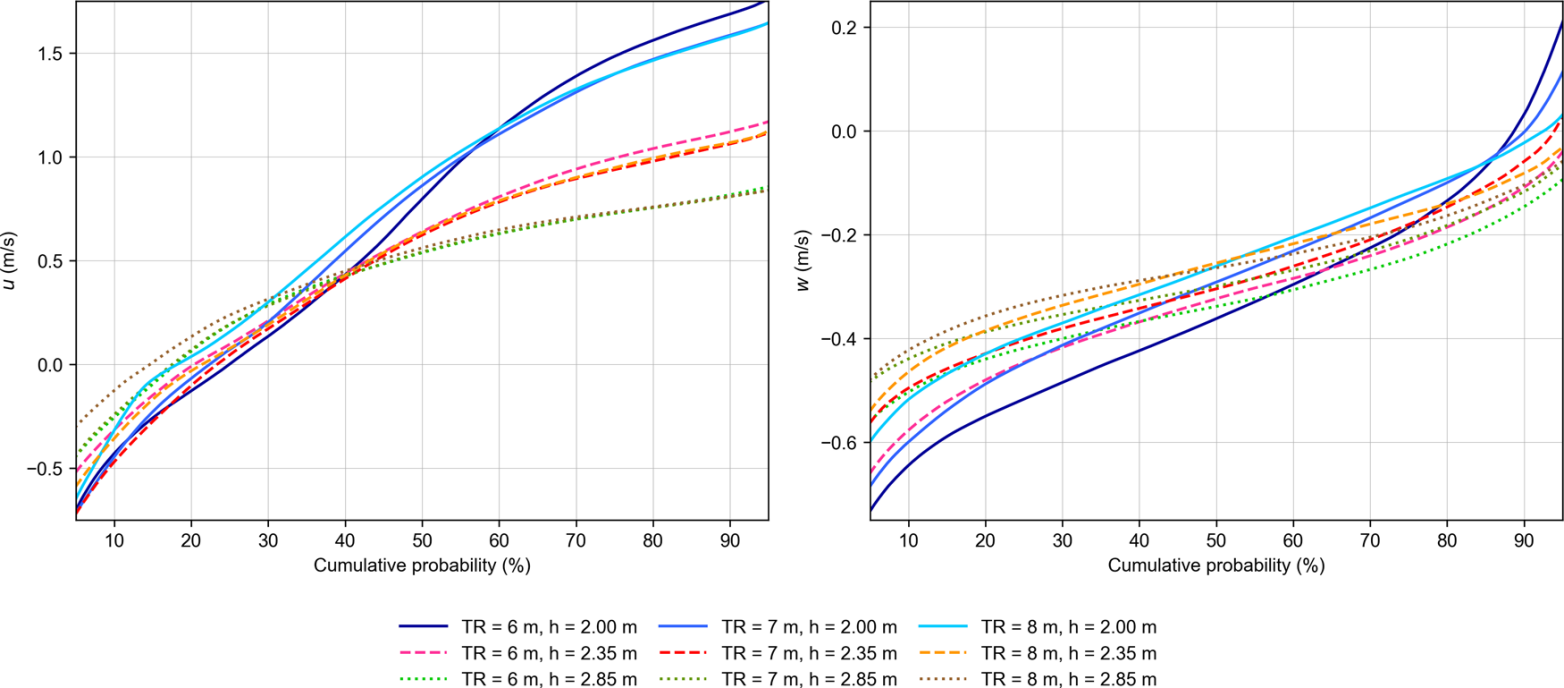
Exceedance probability for streamwise velocity *u* (left) and vertical velocity *w* (right) for the nine production simulations. Note: the x-axis is limited to 5–95% cumulative probability to improve readability

Increasing submergence leads to a systematic reduction in extreme downward-directed velocities at all rack lengths (Table 5; Fig. 7b). For example, at a trash rack length of 6 m, TailMean5 *w* decreases by 25% from −0.83 m/s at 2.00 m submergence to −0.62 m/s at 2.85 m water depth. Comparable monotonic reductions of 33% and 23% are observed for rack lengths of 7 m and 8 m, respectively (Table 5). In general, the spatial distribution of downward velocity cores becomes progressively more uniform with increasing headwater level (Fig. 6).

The ratio of the mean streamwise velocity component to the mean vertical velocity component increases with the length of the rack (Table 5). This trend indicates a relative enhancement in horizontal transport towards the downstream bypass in comparison to downward deflection. Conversely, with increasing submergence, there is a consistent decrease in the *u*/*w* ratio. This occurs because mean *u* also decreases as submergence rises, while mean *w* remains relatively unaffected by changes in submergence.

Free-surface elevation is shown as deviation from the maximum value within each slice (range −0.30 m to 0.0 m) to allow comparison across different submergence levels (Fig. 6). Free-surface variability decreases consistently with increasing submergence. For a trash rack length of 6 m, the interquartile range (FSE_P95–P5) decreases from 0.15 m at 2.00 m submergence to 0.03 m at 2.85 m. This stepwise reduction of 0.12 m is the largest for the 6 m-long trash rack scenarios, but is still observed for the other rack lengths (0.09 m and 0.05 m; Table 5). The same trend is visible when looking at the free-surface elevation standard deviation (FSE_std). Increased submergence and rack length reduce localized surface depressions and homogenize the near-rack velocity field. The lowest submergence configuration (i.e., 6 m trash rack length, 2.0 m submergence) exhibits pronounced localized surface depressions coinciding with concentrated downward-directed velocity cores above the rack. With increasing submergence and rack length, these depressions diminish, and the free surface becomes more spatially uniform. This homogenization of the approach flow corresponds to reduced magnitudes of TailMean5 *w* and lower FSE variability metrics, indicating improved hydraulic stability of the intake region.

The spatial structure of the flow field corresponding to these quantitative trends is illustrated in Fig. 6 for three configurations. In the top row (Configuration 1: TR = 6 m, *h* = 2.00 m), the streamwise velocity slice reveals a concentrated high-velocity band on the orographic left side of the rack, with *u* > 1.0 m/s, while the vertical velocity map shows strongly negative cores, with *w* < −0.5 m/s coexisting with weakly positive regions in the intake plane. The free-surface deviation panel shows pronounced depressions of up to −0.30 m, coinciding spatially with upward velocity cores. In the middle row (Configuration 5: TR = 7 m, *h* = 2.35 m), the streamwise velocity heterogeneity is visibly reduced. The downward cores are less pronounced and more spatially distributed. Also, the free-surface depression detected in configuration 1 is diminished. The flow field in the bottom row (Configuration 9: TR = 8 m, *h* = 2.85 m) is the most uniform of the three configurations, with the free-surface deviation approaching zero across the intake area. With increasing submergence and rack length, both the magnitude and spatial variability of the velocity field decrease, resulting in a more uniform distribution of vertical deflection across the intake. This progressive homogenization observed in Fig. 6 is consistent with the reduction of tail-based velocity metrics (Table 5) and the flattening of the cumulative velocity distributions (Fig. 7).

The quantitative distribution of *u* and *w* within the combined VOI is illustrated by the cumulative probability curves shown in Fig. 7. These curves provide a complementary perspective to the summary statistics in Table 5 by representing the full distribution of velocity values across the intake region. Figure 7a shows that increasing submergence systematically reduces streamwise velocities across nearly the entire probability range, while the influence of trash rack length on *u* remains comparatively small. In contrast, the distribution of vertical velocities (Fig. 7b) reveals that both increasing rack length and increasing submergence progressively flatten the curve, indicating a more homogeneous downward velocity distribution above the rack. Among the two parameters, submergence exerts the stronger influence on this homogenization effect, as reflected by the substantial reduction of extreme downward velocities in the lower tail of the distribution. These distribution-based results are consistent with the reductions in TailMean5 *w* reported in Table 5 and with the spatial patterns observed in Fig. 6.

## Discussion

Previous studies have investigated SHPP prototype configurations (Geiger et al. 2016; Alapfy et al. 2024) and ecological performance (e.g., Knott et al., 2023a; Funk et al., 2024) under specific conditions. However, a structured parameter-based hydraulic optimization framework for SHPP intakes under site-specific geometric constraints is still lacking. The present study applies a previously validated CFD framework (Alapfy et al., 2024) in a comparative analysis to demonstrate that intake hydraulics are governed by the interaction of upstream approach conditions, submergence, and effective intake area. Because all nine configurations were evaluated with the same model setup, the relative differences between cases are robust. The grid-induced uncertainty identified in the grid sensitivity analysis (approximately 5% in TailMean5 *w* between the 60 and 50 mm grids) is small relative to the configuration-to-configuration range observed across the nine cases (57%), further supporting the robustness of these findings.

In the At-Bashy case, the analyzed SHPP module is located at the orographic left side of the existing weir. This geometric constraint produces a distinctly asymmetric approach flow (El Khoury et al. 2010). From a plan-view perspective, the inflow undergoes a triple deflection before reaching the trash rack: the first and second turns are due to the lateral alignment of the module within the river cross-section, the third one due to the downstream turning of the flow toward the intake opening. Consequently, flow separation occurs at the orographic right pier of the weir field, below which the SHPP is installed. This separation likely enhances spatial heterogeneity of the approach flow and contributes to the localized vertical velocity cores observed above the rack.

The CFD modeling results show that increasing submergence reduces approach velocities by enlarging the effective flow cross-section, thereby weakening vertical acceleration toward the rack. This mechanism leads to a consistent reduction in extreme downward-directed velocities and improved free-surface uniformity. Increasing rack length distributes intake loading over a larger area, primarily reducing the average downward velocity. While both parameters improve intake hydraulics, submergence predominantly controls extreme vertical velocity magnitudes and surface variability, whereas rack length primarily influences the mean downward loading.

The streamwise velocity *u* exhibits a guiding effect toward the downstream bypass opening in all analyzed configurations. However, pronounced spatial heterogeneity is visible for configurations with low submergence, where concentrated downward-directed velocity cores coincide with localized surface depressions (Fig. 4).

These hydraulic insights are also key for environmental management, particularly for safely guiding downstream migrating fish over the rack and through the bypass into the tailwater (Knott et al. 2023a). Mean downward flow velocities are below the critical threshold (≤0.5 m/s) when fish may be pressed against the rack (Böttcher et al. 2015; Raynal et al., 2013). However, more ecohydraulics research is needed to evaluate how fish respond to downward currents compared to horizontal inflow velocity for which this threshold was established. In all configurations, there appears to be a guiding effect of streamflow velocity towards the bottom bypass (Fig. 5), with a mean *u* of 0.41−0.75 m/s. Indeed, across all configurations, the ratio between streamwise and vertical velocity components remained greater than unity, indicating that horizontal transport toward the bypass dominates over downward deflection toward the rack. Although this ratio decreases slightly with increasing submergence, the streamwise component remains significantly larger than the vertical component in all evaluated cases, suggesting a generally favorable hydraulic guidance tendency. Still, considering a rack clearance of 15 mm, it cannot be fully ruled out that fish, particularly juvenile and sub-adult life stages, follow the main flow and pass through the rack (Knott et al. 2023b; Ebel 2024). A post-implementation monitoring study aims to assess the ecohydraulics response of fish at the At-Bashy SHPP, focusing on the choice of downstream migratory corridors (i.e., fish pass, bottom outlet, or trash rack/turbine passage).

The comparison between mean values and tail-based metrics highlights the importance of assessing localized extremes rather than relying solely on averaged indicators. Under low-submergence conditions, strongly negative vertical velocities are concentrated in spatially limited cores, while adjacent regions may exhibit weak upward components (Figs. 5, 6). Such heterogeneity implies uneven hydraulic exposure above the rack. The tail-based indicators and spatial visualizations therefore, provide a more comprehensive representation of near-rack flow conditions than mean values alone.

The tail-based indicators further show that increasing rack length from 6 m to 7 m yields a substantial reduction in extreme downward velocities. However, further extension to 8 m produces only marginal additional improvement, particularly at submergence levels of 2.35 m and higher. This indicates diminishing hydraulic returns with increasing intake length. Considering both hydraulic performance and structural-economic implications, a rack length of approximately 7 m represents a balanced solution for the At-Bashy configuration.

Free-surface variability further reflects the spatial stability of the intake hydraulics. Pronounced surface depressions coincide with concentrated downward momentum transfer, whereas reduced variability indicates a more homogeneous and stable approach flow. Although the URANS framework applied in this study does not explicitly resolve transient vortex structures, the consistent reduction of surface variability with increasing submergence and rack length supports the interpretation of improved hydraulic stability.

The results also reveal a hydraulic-ecological and economic trade-off. Increasing submergence improves homogenization of the intake flow but simultaneously increases hydrostatic pressure at the bypass opening. For bottom-located bypass systems, outlet velocities increase with the square root of the effective head according to Torricelli’s law (Hoshins 1907), potentially introducing additional ecological constraints, e.g., posed by strong spatial velocity gradients in the bypass (Enders et al. 2009; Kopecki et al. 2024). From an economic perspective, larger rack lengths require increased concrete volumes, bigger rack modules, and more powerful cleaning mechanisms, while higher submergence necessitates stronger sliding gates, reinforced structural elements, and upgraded drive systems. Ecohydraulic improvements must therefore be balanced against structural and operational costs.

The strong influence of upstream asymmetry observed in this case study suggests that intake optimization cannot be separated from the broader hydraulic setting. For future SHPP implementations, establishing a uniform and symmetric upstream approach flow is recommended wherever feasible, as this may inherently reduce flow separation and near-rack heterogeneity. If geometric or site constraints prevent such alignment, negative hydraulic effects may be mitigated by increasing submergence and/or effective intake area, as demonstrated in the present analysis. However, this may come at the cost of effective fish protection and fish guidance systems.

## Conclusions and Outlook

This study presents a structured three-dimensional CFD-based framework for hydraulic optimization of intake configurations in one-sided shaft hydropower plant (SHPP) layouts, applied within an active engineering design process for the development of the At-Bashy hydropower project in the Kyrgyz Republic. The results demonstrate that increasing submergence and rack length systematically reduce extreme downward-directed velocities and improve free-surface uniformity. However, upstream geometric constraints of the At-Bashy case study strongly influence the spatial distribution of the intake flow.

The analysis confirms that no universally applicable empirical design rules exist for SHPP intake optimization under site-specific boundary conditions. Instead, design decisions must consider hydraulic performance, ecological implications, and economic constraints simultaneously. The applied CFD methodology enables a parameter-driven evaluation of design alternatives and provides a transparent basis for balancing these aspects. The results show that intake hydraulics of SHPP systems can be improved by increasing both the intake area and the submergence above the fine bar rack; however, the ecologically optimal submergence must balance the homogenization of the near-rack flow field with the increasing velocity gradients that may occur at bottom-near bypass openings under higher headwater levels.

The present results should be interpreted within the methodological boundaries of the applied framework. The CFD setup has been validated for this class of shaft hydropower plant intake configuration in Alapfy et al. (2024), and all nine configurations are evaluated comparatively under identical model conditions, ensuring that the relative differences between cases are robust. However, absolute velocity magnitudes carry the residual uncertainties inherent to the numerical model setup. These uncertainties do not affect the ranking and interpretation of configurations but should be considered when applying the reported absolute indicator values beyond the comparative context of this study.

Future work after commissioning of the At-Bashy SHPP will focus on structured field measurements to validate the numerical framework, also under real operating conditions. Such field validation will assess the predictive capability of the near-rack and surface-based indicators defined in this study and further strengthen the applicability of the proposed optimization approach for sustainable SHPP design. Once field-validated, the presented CFD-based framework will support the systematic optimization of vertical intake structures, improving hydraulic performance while maximizing ecological compatibility. Beyond the hydraulic assessment, post-implementation monitoring will assess fish ecological sustainability with a focus on migratory corridors that fish may choose.

The approach illustrates how hydraulic optimization can be integrated with real-world project constraints in an active engineering-design workflow, linking numerical modeling to practical hydropower implementation while meeting ecohydraulically compatible objectives.

## Data Availability

The data presented in this study were generated using three-dimensional CFD simulations. The datasets are available from the corresponding author upon reasonable request.
